# IL-12/IL-23p40 Is Highly Expressed in Secondary Lymphoid Organs and the CNS during All Stages of EAE, but Its Deletion Does Not Affect Disease Perpetuation

**DOI:** 10.1371/journal.pone.0165248

**Published:** 2016-10-25

**Authors:** Petra D. Cravens, Rehana Z. Hussain, William A. Miller-Little, Li-Hong Ben, Benjamin M. Segal, Emily Herndon, Olaf Stüve

**Affiliations:** 1 Department of Neurology, University of Texas Southwestern Medical Center at Dallas, Dallas, TX, United States of America; 2 Department of Neurology, University of Michigan, Ann Arbor, MI, United States of America; 3 Department of Pathology, University of Texas Southwestern Medical Center at Dallas, Dallas, TX, United States of America; 4 Neurology Section, VA North Texas Health Care System, Medical Service, Dallas, TX, United States of America; 5 Department of Neurology, Klinikum rechts der Isar, Technische Universität München, München, Germany; Universitatsklinikum Munster, GERMANY

## Abstract

**Background:**

Interleukin (IL)-12 and IL-23 are heterodimers that share the p40 subunit, and both cytokines are critical in the differentiation of T helper (Th)1 and Th17 cells, respectively. Th1 and Th17 effector cells have been implicated in the pathogenesis of experimental autoimmune encephalitis (EAE), an animal model of the human central nervous system (CNS) autoimmune demyelinating disorder multiple sclerosis (MS). However, ustekinumab, a monoclonal antibody (mAb) against p40 failed to show efficacy over placebo in a phase II clinical trial in patients with MS. The role of p40 in initial T cell priming and maintenance in secondary lymphoid tissues is not yet well understood.

**Methods:**

Active EAE was induced in the B6.129-IL12b strain of p40eYFP reporter mice (yet40 mice), and Th1 and Th17 polarized cells were adoptively transferred into p40-deficient mice. Cellular subsets were phenotyped by multi-parameter flow cytometry, and p40 tissue expression was identified by confocal microscopy.

**Results:**

We show that yet40 mice are susceptible to EAE, and that p40 is highly expressed in secondary lymphoid organs and the CNS during all stages of the disease. Interestingly, p40 expression in the recipient is not required for EAE induction after adoptive transfer of activated and differentiated encephalitogenic Th1 and Th17 cells into p40-deficient mice. Peripheral antagonism of T helper cell trophic factors critical for the differentiation and maintenance of Th1 and Th17 cells ameliorates EAE, indicating that p40 may play a critical role in the induction of CNS autoimmunity but not in its perpetuation.

**Conclusion:**

Our data may explain why ustekinumab did not ameliorate paraclinical and clinical disease in patients with MS. In patients with already established disease, activated antigen-specific encephalitogenic CD4^+^ T cells are likely already differentiated, and are not dependent on p40 for maintenance. A clinical trial of longer duration with anti-p40 mAbs or other forms of pharmacological p40 antagonism, or sequential anti-p40 therapy following T cell depletion may show a benefit by affecting *de novo* generation of autoimmune T cells.

## Introduction

Multiple sclerosis (MS) is an inflammatory demyelinating disease of the central nervous system (CNS) of unknown etiology [1]. It is currently thought that dysregulated CD4^+^ T cell immune responses play a critical role in MS pathogenesis [2]. The role of CD4^+^ T cell subsets in the etiology of MS is substantiated by observations made in the murine model experimental autoimmune encephalomyelitis (EAE) [3]. An early event in the activation and subsequent differentiation of naïve antigen-specific T cells into effector cells in EAE, and possibly MS is directed by their interaction with antigen-presenting cells (APCs) in lymphoid tissues [4]. APCs, including dendritic cells and macrophages, produce interleukin (IL)-12, which induces interferon gamma (IFNγ) secretion and CD4^+^ T helper (Th)1 differentiation. Additionally, these APC can secrete IL-23 that promotes Th17 differentiation [5]. IL-12 and IL-23 are heterodimers that share the p40 subunit: Biologically active IL-12 is comprised of p35p40, whereas IL-23 consists of p19p40. p40 is also secreted as a homodimer [6] that may function as an IL-12 antagonist by competing for the IL-12 receptor and has been shown to induce expression of lymphotoxin-alpha (LTα) and IL-16 in microglia and various other cell types of the immune system. Expression of IL-12 or IL-23 by activated APCs may therefore be considered an early event in the generation of an immune response. Both Th1 and Th17 effector cells have been implicated in the pathogenesis of EAE [7], but the role of p40 in EAE, and specifically in initial T cell priming in secondary lymphoid tissues is not yet well understood, although IL-12p40 mRNA is present in active MS lesions [8]. In addition, the role of p40 in the reactivation of T cells within the CNS has not been studied. It is thought that T cell reactivation and perpetuation of cellular immune responses within the brain and spinal cord are the result of presentation by APCs of CNS autoantigen in cerebral perivascular spaces (CPVS) [9–11], the subarachnoid space [12], interaction with B cells in ectopic germinal centers within the meninges [13], or the presentation of antigen by microglial cells within the CNS parenchyma [14].

In a phase II, multicenter, randomized, double-blind, placebo-controlled study, 249 patients with relapsing-remitting MS (RRMS) received placebo or four different doses of ustekinumab, a monoclonal antibody (mAb) against p40 [8]. Given the strong biological rationale for this trial based on our understanding of the pathogenesis of MS, it was perhaps surprising that ustekinumab treatment at all doses failed to demonstrate a significant reduction in the accumulation of new gadolinium enhancing lesions on serial cranial magnetic resonance images (MRI) through week 23, the primary endpoint of the study.

In this study we assessed the expression of p40 that could contribute to activation, differentiation, and re-activation of CNS antigen-reactive T cells in secondary lymphoid organs and CNS during the preclinical, acute, and chronic stages of EAE using IL-12/IL-23 p40-eYFP knock-in mice [15]. p40 expression was assessed by confocal microscopy, and leukocyte subsets were immunophenotyped by multiparameter flow cytometry to determine which ones express p40. Additionally, disease was induced by active immunization of p40-deficient mice, or adoptive transfer of different CD4^+^ T helper phenotypes into p40-deficient recipient mice.

## Results

### Yet40 mice are susceptible to EAE

**Fig 1:**
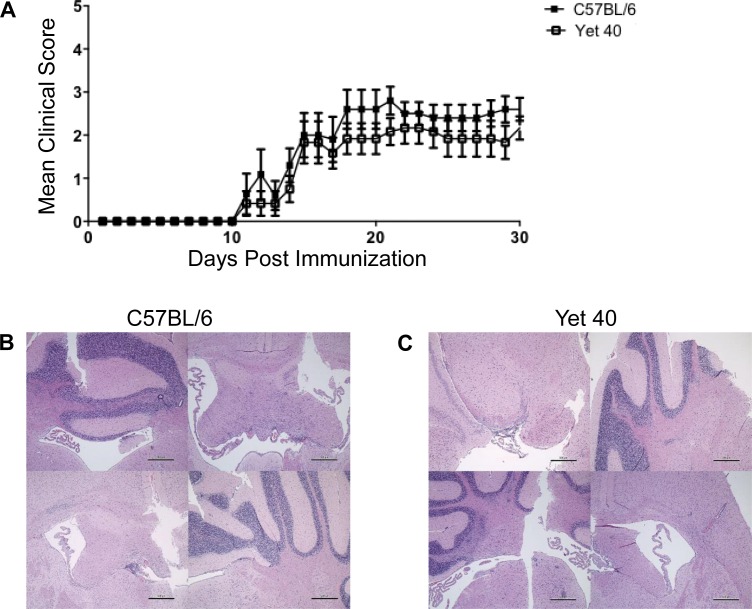
Yet40 mice are susceptible to experimental autoimmune encephalitis (EAE). (A) To identify APC populations that express IL12/23 p40 *in vivo* during EAE, and that are potentially capable of initiating and perpetuating CNS inflammation, B6.129-Il12b strain of p40eYFP reporter mice (yet40 mice) were actively immunized with CFA/MOG_p35-55_. The onset of disease and the clinical disease course were similar in both experimental groups (please see the Material and Methods section for definitions of disease stages). Mean clinical scores of yet40 mice were less severe than those observed in C57BL/6 mice, although those differences were not significant. (B & C) There was no difference in the number of inflammatory cellular infiltrates between yet40 mice and C57BL/6 wild-type mice in the CNS. Representative sections of brain tissue are shown.

To identify APC populations that express IL12/23 p40 *in vivo* during EAE, and that are potentially capable of initiating and perpetuating CNS inflammation, yet40 mice [15] were utilized. Initially, we sought to determine whether yet40 mice differ from C57BL/6 wild-type mice with regard to disease susceptibility, time of disease onset, and disease severity. After active immunization with CFA/MOG_p35-55_, the disease onset of EAE and the clinical disease course were similar in both groups of mice (Fig 1A and Table 1), and differences were statistically not significant. There was no difference in the number of inflammatory cellular infiltrates between yet40 mice and C57BL/6 wild-type mice in the CNS. Representative sections are shown (Fig 1B and 1C).

**Table 1:** Experimental autoimmune encephalitis induced by active immunization.

Group	Incidence	Mean Maximum Score Mean (SD)	Mean Day of Disease onset Mean SD
**C57BL/6**	11/11	3.18 (1.02)	14.54 (3.08)
**Yet40**	10/12	2.42 (1.38)	15.2 (3.46)

Results from a representative experiment is shown.

Abbreviations: Yet40 = B6.129-IL12b strain of p40eYFP reporter mice

### p40 is highly expressed in secondary lymphoid organs during EAE

To investigate which APCs could express IL12/23p40 during the course of EAE, we examined APC subsets from yet40 mice for expression of eYFP using multi-parameter flow cytometry. Because p40 was expected to be expressed by unique APC subsets during different EAE disease stages after active immunization with CFA/MOG_p35-55_, splenocytes were assessed during the pre-clinical, acute, and chronic stages of EAE (please see in the Material and Methods section for definitions of disease stages). In lymph nodes cells, p40 expression was only assessed during the induction and acute disease stages, as we have been unable to detect a substantial degree of antigen presentation in that compartment during the chronic disease stage in this EAE model (data not shown).

**Fig 2:**
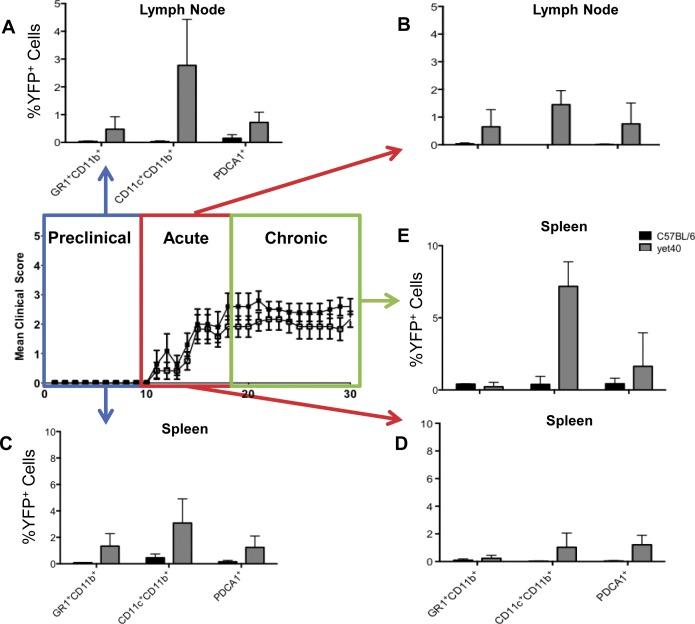
P40 is highly expressed in secondary lymphoid organs during experimental autoimmune encephalitis (EAE). To investigate which APCs could express IL12/23p40 during the course of EAE, we examined APC subsets from B6.129-Il12b strain of p40eYFP reporter mice (yet40 mice) for expression of eYFP by multi-parameter flow cytometry. Splenocytes were assessed during the pre-clinical, acute, and chronic stages of EAE (please see the Material and Methods section for definitions of disease stages). In lymph nodes cells, p40 expression was only assessed during the induction and acute disease stages, as p40 was undetectable during the chronic disease stage in this EAE model (data not shown). (A & B) p40 eYFP expression was readily detectable by flow cytometry in lymph node cells, and (C-E) splenocytes from yet40, but not in control C57BL/6 mice during the pre-clinical, acute, and chronic EAE stages. (A-E) During most disease stages in both compartments, CD11c^+^Cd11b^+^ dendritic cells (DC) were the main expressers of p40-eYFP, followed by PDCA1^+^ plasmacytoid dendritic cells (pDCs), and GR1^+^CD11b^+^ immature myeloid cells.

As predicted, p40 eYFP expression was readily detectable by flow cytometry in lymph node cells (Fig 2A and 2B), and splenocytes (Fig 2C–2E) from immunized yet40 mice, but not in immunized control C57BL/6 mice during the pre-clinical, acute, and chronic EAE stages. During most disease stages in both compartments, CD11c^+^CD11b^+^ dendritic cells (DC) were the main expressers of p40-eYFP, followed by PDCA1^+^ plasmacytoid dendritic cells (pDCs), and GR1^+^CD11b^+^ immature myeloid cells.

### p40 is expressed during all stages of EAE in the brain and spinal cord

**Fig 3:**
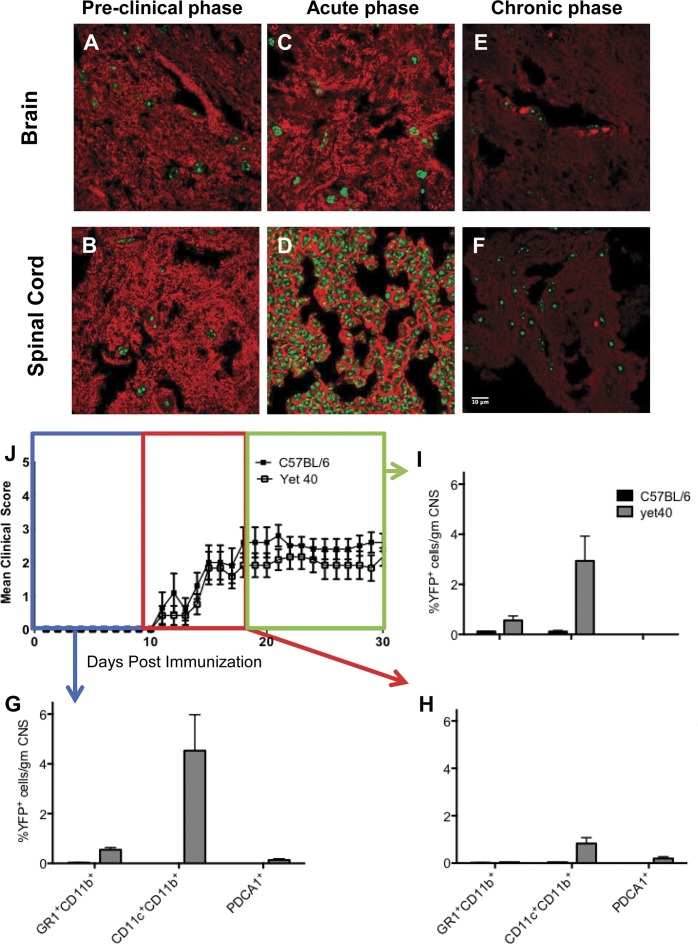
p40 is expressed during all stages of experimental autoimmune encephalitis (EAE) in the brain and spinal cord. (A & B) p40 was detectable in the brain and spinal cord by confocal microscopy during the pre-clinical stage of EAE, (C & D) the acute stage of EAE, and the (E & F) chronic stage of EAE by confocal microscopy (please see in the Material and Methods section for definitions of disease stages). Expression of p40 was highest during the acute EAE stage in the spinal cord. (G) When pooled cells from brain and spinal cord were immunophenotyped by multi-parameter flow cytometry, the highest cellular expressers of p40-eYFP during the pre-clinical stage of EAE, (H) the acute stage of EAE, and the (I) chronic stage of EAE were CD11c^+^Cd11b^+^ DC, followed by GR1^+^CD11b^+^ immature myeloid cells during the pre-clinical stage, and the acute stage. (G-I) Interestingly, the percentage of PDCA1^+^ plasmacytoid DC increased as the disease progressed and decreased in severity.

We were able to detect p40 in the brain and spinal cord by confocal microscopy during the pre-clinical stage of EAE (Fig 3A and 3B), the acute stage of EAE (Fig 3C and 3D), and the chronic stage of EAE by confocal microscopy (Fig 3E and 3F). Expression of p40 was highest during the acute EAE stage in the spinal cord. When pooled cells from brain and spinal cord were immunophenotyped by multi-parameter flow cytometry, the highest cellular expressers of p40-eYFP during the pre-clinical stage of EAE (Fig 3G), the acute stage of EAE (Fig 3H), and the chronic stage of EAE (Fig 3I) were CD11c^+^CD11b^+^ DC, followed by GR1^+^CD11b^+^ immature myeloid cells during the pre-clinical stage, and the acute stage. Interestingly, the percentage of PDCA1^+^ plasmacytoid DC increased as the disease progressed and decreased in severity (Fig 3G–3I).

The frequency of p40^+^ cells in all compartments during all disease stages was less than 10%. We believe that this is a true result. Reinhardt et al provided confirmation that the knockin allele in yet40 mice is a reliable reporter [15]. On a per-cell basis, these investigators demonstrated by intracellular cytokine staining that comparable numbers of bone marrow-derived DC from p40 (^+/-^) and p40 (^y/-^) mice expressed p40, corroborating faithful p40 production from the knockin allele. Although a small percentage of cells in p40 (^y/-^) mice were p40^+^ and eYFP^-^, kinetic analysis revealed that all p40^+^ cells eventually became eYFP^+^, which was interpreted as a delay in folding of eYFP to a fluorescent-competent state. Thus, it is conceivable that confocal microscopy analyses at later time points may have resulted in a higher percentage of p40^+^ myeloid cells.

### p40 expression in the brain is not required for EAE induction after adoptive transfer of encephalitogenic T cells

**Fig 4:**
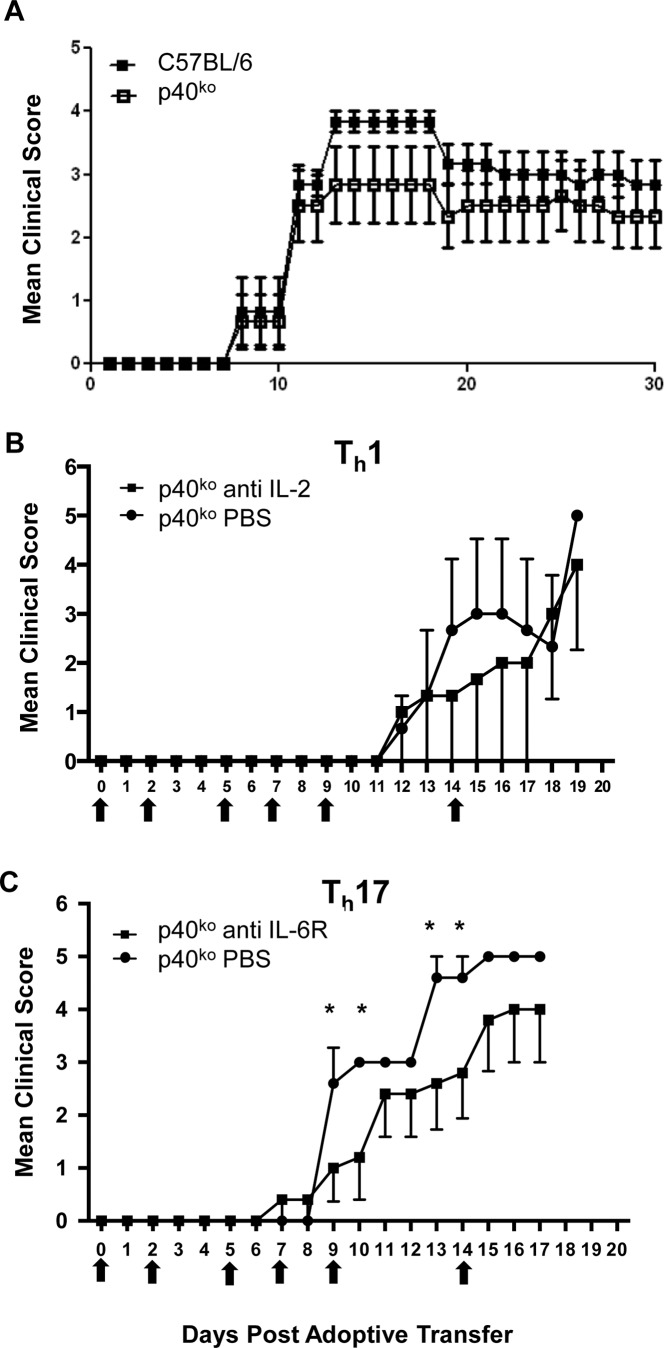
p40 expression in the brain is not required for experimental autoimmune encephalitis (EAE) induction after adoptive transfer of encephalitogenic T cells. (A) Adoptive transfer of CD4^+^ T cells from C57BL/6 donor mice into C57BL/6 recipient mice, or into p40-deficient (p40^ko^) mice resulted in EAE, indicating that p40 expression in the CNS is not an absolute requirement for CNS autoimmunity to occur once antigen-specific peripheral CD4^+^ T cells are activated and differentiated. (B & C) When CD4^+^ T cells from 2DD donor mice were polarized into Th1 cells or Th17 cells, transfer of either phenotype into p40^ko^ mice also resulted in severe EAE. (B) Peripheral antagonism of IL-2, a trophic factor required for proliferation and differentiation of Th precursor cells into effector Th1 cells with a monoclonal antibody resulted in slightly milder disease during the acute stage of Th1-mediated adoptively transferred EAE. IL-6 is a cytokine critical for the differentiation and maintenance of Th17 cells, and (C) peripheral antagonism of IL-6R with a monoclonal antibody also decreased the severity of EAE substantially during the acute stage of Th17-mediated adoptively transferred EAE.

Adoptive transfer of CD4^+^ T cells from C57BL/6 donor mice into C57BL/6 recipient mice, or into p40^ko^ mice resulted in EAE (Fig 4A), indicating that p40 expression in the CNS is not an absolute requirement for CNS autoimmunity to occur once antigen-specific peripheral CD4^+^ T cells are activated and differentiated.

### Adoptive transfer of encephalitogenic Th1 and Th17 cells cause EAE in p40-deficient mice

When CD4^+^ T cells from 2D2 donor mice were polarized into Th1 cells or Th17 cells, transfer of either phenotype into p40^ko^ mice resulted in severe EAE (Fig 4B and 4C; Table 2).

**Table 2:** Experimental autoimmune encephalitis induced by adoptive transfer.

Group	Incidence	Mean Maximum Score Mean (SD)	Mean Day of Disease onset
**C57BL/6**	4/4	3.75 (0.43)	10.25 (1.29)
**p40**^**ko**^	3/4	3 (1.73)	10 (1.41)
**Th1 PBS**	3/3	4.33 (0.74)	15.33 (2.62)
**Th1 anti-IL-2**	2/3	2 (1.63)	15 (3)
**Th17 PBS**	5/5	5 (0)	9.2 (0.4)
**Th17 Anti IL-6**	4/5	3.8 (1.94)	9.5 (1.66)

Results from a representative experiment is shown.

Abbreviations: ko = deficient; Th = T helper; IL = interleukin; PBS = phosphate buffered saline

### Peripheral antagonism of T helper cell trophic factors ameliorates EAE

IL-2 is the predominant cytokine produced by naive Th cells in a primary response. It is required for proliferation and differentiation of Th precursor cells into effector cells, including Th1 cells [16]. The role of IL-6 in Th17 cell differentiation is critical, as Th0 cells require an environment that is high in IL-1, IL-6, IL-23, and TGF-β [17] to do so. Th17 cells in turn induce various downstream cytokines and chemokines, including IL-6, [18–20], IL-8, and G-CSF [21]. IgG antibodies have very poor CNS bioavailability. The ratio of IgG concentration in the brain relative to plasma is in the range of 1:500 [22], and therefore it has to be assumed that all major direct biological effects of therapeutic mAb occur in the periphery. Peripheral antagonism of IL-2 with a monoclonal antibody resulted in slightly milder disease during the acute stage of Th1-mediated adoptively transferred EAE (Fig 4B and Table 2), and peripheral antagonism of IL-6R with a monoclonal antibody also decreased the severity of EAE substantially during the acute stage of Th17-mediated adoptively transferred EAE (Fig 4C and Table 2).

## Discussion

Two EAE models were utilized to examine the role of p40 in the initiation and perpetuation of CNS autoimmunity. In mice that have been actively immunized, the CFA/MOG_p35-55_ emulsion supplemented with mycobacterium leads to activation of macrophages and DC. Antigen-loaded APC exit the skin and traffic to the draining LN where they present MOG peptide to naïve T cells. This interaction results in activation and clonal expansion of MOG_p35-55_-specific T cells, some of which enter the brain and spinal cord and cause clinical EAE by day 12–14. In the active EAE models, other investigators demonstrated in mice genetically deficient in p40 that this molecule is an absolute requirement for disease induction [23, 24]. Our own active EAE experiments show that p40 is expressed in secondary lymphoid organs, the brain, and the spinal cord of experimental animals during all clinical stages of EAE by different myeloid cell subsets.

In contrast to the active EAE model, in passive EAE, or the adoptive transfer EAE model, activated and differentiated CD4^+^ T helper cells from a donor animal are transferred to a naïve syngeneic recipient. Thus, the requirement of activation and differentiation of CD4^+^ T helper cells in the recipient is no longer required. However, activated encephalitogenic donor cells require trophic factors for their maintenance and expansion. Our experiments show that p40 is very likely not required for EAE disease susceptibility in the adoptive transfer model.

However, a limitation of the experimental approach that was chosen is the disease kinetic of our adoptive transfer EAE model. The disease course develops rapidly, is severe, and mostly monophasic [25]. Transferring encephalitogenic T cells in excess may mask the function that IL-23 plays in the maintenance of encephalitogenic Th 17 cells in MS.

Other investigators demonstrated that the presence of myeloid cells in the CPVS is required to induce EAE [9–11]. Requirement for these cells has been thought to be associated with their ability to process and present antigen [26]. Furthermore, these cells produce cytokines that are necessary for T cell survival. We show that both, Th1 and Th17 differentiated CD4^+^ T cells can induce EAE in the absence of p40, elaborating on data by Grifka-Walk et al [27]. We also demonstrate that antagonism of trophic factors that interfere with the maintenance of Th1 and Th17 cells in the periphery can still ameliorate disease in that situation.

In MS, it is currently unknown whether an aberrant Th cell reactivity is always directed against the same antigen, or whether these responses diversify during sequential clinical attacks or disease progression. A typical immune response against a protein is focused on one or two dominant epitopes. Epitope spreading is defined as the diversification of epitope specificity cellular adaptive immune responses from the initial dominant epitope to subdominant, or cryptic epitopes [28]. This propagation of cellular adaptive immune responses from dominant to cryptic epitopes may be the result of differential protein processing or antigen presentation by different APC populations, and also to the availability of epitope-specific T cells [29]. While epitope spreading has not been conclusively demonstrated in MS or many other human autoimmune disorders, it is conceivable that a trial with ustekinumab of sufficient duration to interfere with the generation of Th1 and Th17 cells that recognize previously cryptic determinants as dominant could decrease the frequency of clinical relapses and the accumulation of new CNS lesions.

In summary, our observations indicated that p40 may play a critical role in the induction of CNS autoimmunity, but not in its perpetuation. Our data may explain why ustekinumab, a mAb against p40 did not ameliorate paraclinical and clinical disease in patients with MS during a relatively short clinical trial [8]. In patients with already established disease, activated antigen-specific encephalitogenic CD4^+^ T cells are likely to have already differentiated, and many of these cells may not dependent on p40 for maintenance.

## Material and Methods

### Mice

B6.129-Il12b strain of p40eYFP reporter mice (yet40 mice), p40-deficient mice (p40^ko^ mice), and MOG_p35-55_ T cell receptor (TCR) transgenic (Tg) mice (2D2 mice) [30] were purchased from Jackson Laboratories (Bar Harbor, ME, USA). Yet40 mice are homozygous for a bicistronic yet40 allele in which an IRES-eYFP is inserted downstream from the translational stop codon [15]. This construct allows for normal expression of p40 with the concomitant expression of eYFP. C57BL/6 mice were bred at UT Southwestern. Mice were used at 8–12 weeks of age. Animals were housed in a specific pathogen-free facility at UT Southwestern. All breeding and experiments were reviewed and approved by the Institutional Animal Care and Use committee at UT Southwestern permit number: 2008–0032).

### Peptides

Myelin oligodendrocyte glycoprotein (MOG) peptide (p) 35–55 was synthesized by S.C. Bio (Menlo Park, CA, USA)

### Antibodies

Cells were stained with the following mAb: Anti-CD4-PE Texas Red (RM4-5, Invitrogen, Carlsbad, CA, USA), anti-CD11C-APC or anti-CD11c-PE (HL3, BD Biosciences, San Jose, CA, USA), anti-CD19- Alexa Fluor 700 (eBioID3, eBioscience, San Diego, CA, USA), anti-CD3-Pacific Blue (500A2, BD Biosciences), anti-CD11b-PerCP-Cy5.5 (M1/70, eBioscience), anti-MHCII-PE-Cy5, anti-CD45-PE-Cy7 (30-F11, eBioscience), anti-GR-1-APC-Cy7 (RB6-8C5, BD), anti-CD8-Pacific Orange, and anti-CD103-APC, CD115-APC (AF598, eBioscience), anti-PDCA-1-Biotin (JF05-1C2.4.1, Miltenyi Biotec, Bergisch Gladbach, Germany), anti-B220-PE (RA3-6B2, Biolegend, San Diego, CA, USA), and anti-DX5-biotin (Biolegend). Biotinylated mAb were revealed with Streptavidin-Q Dot 655 (Thermo Fisher Scientific, Waltham, MA, USA).

For *in vivo* adoptive transfer experiments, the following mAb were utilized: Anti- murine IL-2 (JES6-1A12, Biolegend), and anti- murine IL6R (Genentech Inc., South San Francisco, CA, USA).

### Induction of EAE by active immunization

C57BL/6 mice and Yet40 mice were anesthetized with tribromoethanol (^®^Avertin; Sigma Aldrich, St. Louis, MO, USA) 250 mg/kg intraperitoneally (i.p.) followed by subcutaneous (s.c.) immunization with 200 μg/100 μL CFA/ MOG_p35-55_ supplemented with 400 μg/ml of mycobacterium tuberculosis H37RA (MT; DIFCO Laboratories, Detroit, MI, USA) at the flanks. Mice were injected i.p. 200 ng/200 μl of with pertussis toxin (PT) at days 0 and 2. Animals were monitored daily for development of EAE and scored for disease as follows: 1, limp tail or hind limb weakness, but not both; 2, limp tail and hind limb weakness; 3, moderate hind limb weakness with or without unilateral hind limb paralysis; 4, bilateral complete hind limb paralysis; 5, complete hind limb paralysis with forelimb weakness; moribund state.

The clinical course of EAE was divided into three disease stages. The induction stage commenced at the time of active immunization with CFA/ MOG_p35-55_. The active disease stage started at the onset of clinical disease. The chronic disease stage began once the disease severity reached its peak and plateaued.

Observation of all experimental animals occurred at least twice daily upon disease reaching a score of 3, with documentation of the clinical score. The following interventions were cumulative: Once a mouse reached clinical score 2, moist chow was provided daily. At score 3, animal weights were recorded daily, and animals were euthanized if the weight loss was greater than 20% from baseline. When mice scored 4, the urinary bladder was palpated and manually expressed as needed, and affected animals were no longer housed with cage mates of a lower score. Furthermore, suitable nesting material was always being provided. Affected mice had to be euthanized if there was no improvement after 72 hours at score of 4. Animals were euthanized immediately upon observation of a score of 5, regardless of time to development. Euthanasia was performed by carbon dioxide narcosis. Cervical dislocation was always used as a secondary physical method. Death was confirmed by observation of a lack of breathing, loss of heartbeat, glazed appearance to the eyes, and loss of limb movement.

### Induction of EAE by adoptive transfer of activated CNS antigen specific T cells

EAE by adoptive transfer of C57BL/6 donor cells was induced as described previously [31]. Briefly, wildtype C57BL/6 donor mice were actively immunized for EAE as described above. On day 12 post immunization the mice were euthanized and the subiliac and axillary lymph nodes collected. Single cell suspensions were prepared by pressing the lymph nodes through 70 μm mesh. Cells were cultured in RPMI 1640 (Corning, Cellgro) supplemented with 10% v/v FBS (Invitrogen), 2mM Glutamine, 100 μg/ml penicillin and streptomycin, 10 mM HEPES buffer, 1mM sodium pyruvate, MEM NEAA (Corning, Cellgro), and 0.5μM 2-mercaptoethanol (Fisher Scientific, Waltham, MA, USA). Lymph node cells were also cultured in the presence of 10 μg/mL of MOG_p35-55_, and 0.5 ng/mL IL-12 (R&D Systems, Minneapolis, MN, USA). On day 3 of culture, cells were collected, washed, counted and 10 X10^6^ cells injected i.p. to recipient C57BL/6 and p40^ko^ mice. Animals were monitored daily for development of EAE and scored as described above.

For adoptive transfer of CD4^+^ T cells from 2D2 mice, a modified version of a published protocol was utilized [32]. Briefly, spleens and lymph nodes were isolated from naive 6–10 week old mice, and single-cell suspensions were made by passing through a 70 μm nylon cell strainer (BD Biosciences. CD4^+^ T helper cells were isolated from splenocytes by negative selection using the EasyStep Mouse CD4^+^ T cell Isolation Kit (Stem Cell Technologies, Vancouver, BC, Canada), following the manufacturers protocol. Cells were cultured in RPMI 1640 (Corning, Cellgro). Supplemented cells were cultured in RPMI 1640 (Corning, Cellgro) supplemented as described above.

For Th17 polarization, cells were cultured in complete RPMI and activated with irradiated C57BL/6 splenocytes and 20 μg/ml MOG_p35–55_ peptide in the presence of 10 ng/ml IL-1β, 30 ng/ml IL-6 (Biolegend), 3 ng/ml TGF-β (R&D Systems), 20 μg/ml anti-IFN-γ (XMG1.2, Biolegend), and anti-IL-4 (11B11, BioXcell, Lebanon, NH, USA) for 72 hours. Cells were then split in half with the addition of new media and 20 ng/ml IL-23 (Biolegend) and further cultured for 96 hours. Live cells were separated by Ficoll^®^ gradient (Lymphocyte Separation Medium, GE Healthcare Life Sciences, Marlborough, MA, USA), and re-stimulated with anti-CD3 (145-2C11, Bio X cell) and anti-CD28 (37.51, BioXcell) for 72 hours.

For Th1 polarization, cells were cultured in complete RPMI and activated with irradiated C57BL/6 splenocytes and 20 μg/ml MOG_p35–55_ in the presence of 10 ng/mL IL-12 (R&D Systems) for 72 hours. Cells were then split in half with the addition of new media and 5 ng/ml IL-2 (Biolegend) and further cultured for 96 hours. Live cells were separated by Ficoll gradient (Lymphocyte Separation Medium, GE Healthcare Life Sciences), and re-stimulated with anti-CD3 (145-2C11, Bio X cell) and anti-CD28 (37.51, Bio X cell) for 72 hours.

### Flow Cytometry

At the indicated time points, mice were anesthetized and perfused with ice cold PBS through the left ventricle. Spleens, LN, brains and spinal cords were collected and pressed through 70 μm mesh. Brain and spinal cords cell suspensions were washed twice in 37% Percoll^TM^ (GE Healthcare Bio-Sciences, Pittsburgh, PA, USA) and mononuclear cells isolated by centrifugation of a 30%/70% Percoll^TM^ gradient as previously described [33]. To examine the frequency of immune cell populations in the mice, cells were stained and then fixed in 1% paraformaldehyde (PFA) (Electron Microscopy Sciences, Hatfield, PA, USA). Cells (up to 500,000 events) were collected on a BD FACSAria SORP (BD Biosciences) equipped with 488 nm, 532 nm, 405 nm and 633 nm lasers. Data was analyzed with Flowjo Software (Treestar, Ashland, OR, USA).

### Histology

Following fixation in 10% buffered formalin, coronal sections of brain tissue, axial sections of spinal cord, and longitudinally-oriented optic nerves were processed and embedded in paraffin blocks. 4 μm sections were cut, mounted on Fisher Brand Superfrost Plus glass slides (Fisher Scientific, Pittsburgh, PA), and stained with hematoxylin & eosin (Fisher Scientific). Tissues were scored by a blinded examiner.

### Confocal microscopy

Following fixation in 4% paraformaldehyde for at least 2 hours, tissues were stored in 2% sucrose at 4°C until cutting. The brains were coronally sectioned, and after embedding in Tissue-Tek OCT Compound, the tissues were snap frozen in liquid nitrogen. Two 6 μm-thick sections were cut from each brain with a freezing microtome and mounted on Fisherbrand Superfrost Plus glass slides (Fisher Scientific, Pittsburgh, PA, USA). Tissue sections were then fixed in 10% formalin and rinsed in tap water. One set of tissues was stained with H&E and prepared for light microscopy, and the other set was prepared without staining for fluorescent microscopy, using anti GFP Alexa Fluor 488-conjugate (Invitrogen), and Hoechst 33342 (Invitrogen) for counter-staining. Areas of interest were captured on digitized images using a Leica TCS SP5 confocal microscope with the 63× objective and analyzed by ImageJ 1.35s software (public domain; http://rsbweb.nih.gov/ij/).

### Statistical Analysis

For parametric tests, data were checked for normality by using the Kolmogorov–Smirnov test. Normally distributed values were compared using the unpaired two-sided Student *t*-test. Correlations between continuous and categorical variables were assessed using the Mann-Whitney U-test. All experiments were repeated at least twice. All statistical tests were 2-sided and p < 0.05 indicated significance. All analyses were performed with Prism 5 (Graphpad, La Jolla, CA, USA).

It is generally accepted that 10 mice per treatment group are required to test the effect of reagents in active immunization EAE. This number was determined through power analysis. Through testing the null hypothesis (that the mean disease severity will be equal among the two treatment groups), it is possible to attain >80% power with the following assumptions: The criterion for significance (alpha) has been set at 0.050; the test is two-tailed, which means that an effect in either direction will be interpreted; this computation assumes that the mean difference is 1.7 (corresponding to mean EAE scores of 1.7 versus 0.0; or 2 versus 3.7) and the common intra-group SD is 1.0. This minimum significant difference of 1.7 was selected from prior observations that repeatable effects were associated with changes in mean disease score of between 1.5 and 2.0. Thus, this work assumes that effects smaller than 1.7 would not be of clinical or substantive significance and that differences in mean clinical score of 1.7 can be anticipated. A second goal of this study was to estimate the mean difference between the two populations. On average, a study of this design would enable us to report the mean difference with a precision (95.0% confidence level) of plus/ minus 1.13 points. For example, an observed difference of 1.7 between treatment groups would be reported with a 95.0% CI of 0.57 to 2.83. The precision estimated here is the median precision. Precision will vary as a function of the observed SD (as well as sample size), and in any single study will be narrower or wider than this estimate.
